# Tell Me Who You Vote for, and I'll Tell You Who You Are? The Associations of Political Orientation With Personality and Prosocial Behavior and the Plausibility of Evolutionary Approaches

**DOI:** 10.3389/fpsyg.2021.656725

**Published:** 2021-05-19

**Authors:** Thomas Grünhage, Martin Reuter

**Affiliations:** ^1^Department of Psychology, University of Bonn, Bonn, Germany; ^2^Center for Economics and Neuroscience, University of Bonn, Bonn, Germany

**Keywords:** political orientation, conservatism, behavioral economics, personality, cooperativeness, trust

## Abstract

Blatantly observable in the U.S. currently, the political chasm grows, representing a prototype of political polarization in most if not all western democratic political systems. Differential political psychology strives to trace back increasingly polarized political convictions to differences on the individual level. Recent evolutionary informed approaches suggest that interindividual differences in political orientation reflect differences in group-mindedness and cooperativeness. Contrarily, the existence of meaningful associations between political orientation, personality traits, and interpersonal behavior has been questioned critically. Here, we shortly review evidence showing that these relationships do exist, which supports the assumption that political orientation is deeply rooted in the human condition. Potential reasons for the premature rejection of these relationships and directions for future research are outlined and implications for refinements and extensions of evolutionary informed approaches are derived.

## Introduction

### The Two-Fold Benefits of Differential Political Psychology

Besides shedding light on social aspects as political communication and intergroup-relations, political psychology also encompasses a differential perspective treating a person's political orientation or “ideology” as a trait with potential explanatory power for experience and behavior in- and outside of the political sphere (Jost, 2017; Claessens et al., 2020). Such a conceptualization promises benefits in two directions: First, people's political orientation, often exhibited strongly and willfully, may provide psychologists with hints to the person's personality structure and behavioral inclinations. Second, thorough definitions of the psychological foundations of political orientation enhance the comprehensibility of political phenomena, ranging from the intriguing ideological consistency of issue stances and voting intentions (Jost, 2006) to only superficially logical associations between political orientation and phenomena as environmentalism (Feinberg and Willer, 2013; Wolsko et al., 2016).

However, critics suggested that personal, interpersonal, and political temperaments are largely independent of each other and—though substantially heritable in each case—thus represent distinct dispositions. Those dispositions are thought to influence experience and behavior specifically on the personal, the immediate social (e.g., dyadic), and the large social scale, respectively, but hardly possess any predictive power *across* those categories (Alford and Hibbing, 2007). To clarify this issue, here, we summarize research examining political orientation's associations with personality and interpersonal behavior in controlled experiments and attempt a tentative integration within an evolutionary framework.

### The Two-Fold Nature of Political Orientation

Psychological accounts of political orientation are ample (Jost et al., 2003; Graham et al., 2009; Jost, 2017), but many of them share a structural dualism differentiating two factors. For example, according to the Dual-Process-Model of Ideology (Duckitt and Sibley, 2010), political orientation can be traced back to two persistent motivational goals, namely *Right-Wing-Authoritarianism and Social-Dominance-Orientation*, which are driven by views of the world as a dangerous place or a competitive jungle, respectively. People scoring high on RWA can be characterized as sticking to tradition, being obedient and submissive to authorities, and aggressive toward elements threatening the established order. SDO refers to whether one prefers intergroup relationships to be more hierarchical or more equal.

Another dualistic model relates different forms of moral foundations to political orientation. Specifically, Moral Foundations Theory (MFT) posits five psychological systems, entailing specific emotional reactions that evolved as adaptions to specific evolutionary problems. Whereas, the Harm and Fairness system are referred to as *individualizing* foundations because of their emphasis on individual rights, the Loyalty, Authority and Purity foundations are referred to as *binding* foundations serving group-cohesion (Graham et al., 2009). Political conservatives or right-wingers were shown to endorse RWA, SDO, and binding relative to individualizing foundations more strongly in numerous studies (e.g., Koleva et al., 2012; Grünhage and Reuter, 2020b).

Claessens et al. (2020) intriguingly related this two-fold structure of the psychological underpinnings of political orientation to two significant shifts in human evolution: cooperation across wider networks and increasing group-mindedness. If political orientation traces back to evolutionary evolved psychological mechanisms, there is no reason to assume that experience and behavior outside of the political sphere (or the large social scale) are independent of political orientation. On the contrary, if the claimed independence of personal, interpersonal, and political dispositions were supported, the approach of Claessens et al. (2020) would be rendered implausible.

## Political Orientation and Basic Personality Traits

Though of generally moderate size, meaningful negative associations of conservative political orientation and Big Five traits Openness and Agreeableness, as well as a positive relationship between conservatism and Conscientiousness, are well-established: In high-powered studies across several countries, Openness is consistently negatively associated with conservatism while Conscientiousness is positively (Sibley et al., 2012; Bakker, 2017; Fatke, 2017; Furnham and Fenton-O'Creevy, 2018; Krieger et al., 2019; Grünhage and Reuter, 2020b). Expectably, Openness tends to correlate negatively with measures of social and economic conservatism, while Conscientiousness is more predictive of social than economic conservatism (Carney et al., 2008; Gerber et al., 2010, 2011; Vecchione et al., 2011; Fatke, 2017). The opposite is the case for Agreeableness, which—if at all—predicts support for social justice and redistributive policies predominantly (Gerber et al., 2011; Kandler et al., 2012; also see Sibley and Duckitt, 2008). Accordingly, the effect of Openness on political orientation measures is reflected in equally strong associations with RWA and SDO. Contrarily, Conscientiousness and Agreeableness are more strongly associated with RWA and SDO, respectively (Grünhage and Reuter, 2020b; also see Perry and Sibley, 2012).

In evaluating these and a multitude of less recent studies on personality's association with political orientation, several caveats emerge, the first of which refers to dimensional complexities on both sides of the relationship: As early as 2011, Gerber et al. identified coarse measurements of political orientation as a potential reason for missing or attenuated relationships (Mehrabian, 1996; Alford and Hibbing, 2007). Employing measures differentiating between social and economic conservatism, i.e., acknowledging the two-fold nature described above, led to profound results as in most studies described above (but see Furnham and Fenton-O'Creevy, 2018; Krieger et al., 2019). A valuable qualification of this approach was recently presented by Johnston and colleagues (Johnston's et al., 2017; Johnston, 2018): Using extensive American survey and experimental data, the authors intriguingly showed that measures of closed personality—including but not restricted to Big Five traits—are primarily related to the social or cultural domain of political orientation. Critically, an ideological “alignment” only occurs among politically engaged individuals, i.e., dispositionally closed individuals support conservative social *and* economic policies when they are politically engaged, while—among the unengaged—personality's associations with economic preferences are absent or even reversed. Thus, a top-down (or elite-driven) attitude organization combining conservative cultural and right-wing economic stances seemingly competes with a bottom-up organization combining conservative cultural and *left-wing* economic attitudes. In line with that, Malka et al. (2019) showed that in the majority of 99 examined nations, the social and cultural domain are un- or even negatively correlated. Hence, as social issues dominate our understanding of the political dimension (see Ellis and Stimson, 2012) and serve as cultural cues shaping engaged citizen's economic preferences (Johnston's et al., 2017), distinct predictors of economic preferences may be concealed (Feldman and Johnston, 2014) but may also dilute associations of personality measures with undifferentiated (socially dominated) measures of political orientation. Both was demonstrated most recently by Bakker and Lelkes (2018) and Xu et al. (2021): Here, Extraversion was shown to be associated with economic (but not cultural) conservatism and Agreeableness paradoxically showed positive associations with measures of cultural but strong negative associations with measures of economic conservatism. In line with Johnston, Lavine and Federico (2017)'s argument, however, associations with a general, broad measure of political orientation were driven by the traits predicting social conservatism.

Turning to the other side of the relationship, undifferentiated measurements of personality pose a severe problem, too. In a dedicated research program, Bakker and Lelkes (2018) compared elaborate measures of Big Five traits to a frequently used short version with regard to both' associations with proper measures of political orientation and its two dimensions. With the exception of Neuroticism, the short measure yielded massively attenuated effect sizes, even hiding the mere significance of effects as a positive association of Extraversion and economic conservatism. Of note, such a pattern had also been described in a prior meta-analysis (Sibley et al., 2012) and specific item selection did not account for the attenuation. Xu et al. (2021) even applied instruments allowing for the differentiation of aspects of the Big Five (a theoretical level between the main domains and the constituent facets; DeYoung et al., 2007; Soto and John, 2017) and found associations between almost all of those and properly measured dimensions of political orientation. Notably, differential associations of those aspects were shown to cancel each other out, for example in the case of Compassion vs. Politeness representing aspects of Agreeableness. In line with that, studies using the HEXACO-framework differentiating Agreeableness and Honesty-Humility, found substantial associations with SDO for the latter but not the former (Lee et al., 2010; Sibley et al., 2010). Furthermore, significant associations were found, for example, for the Assertiveness aspect of Extraversion and the Withdrawal aspect of Neuroticism with conservatism and liberalism, respectively (Xu et al., 2021).

Hence, it seems justified to state, that associations between political orientation and Big Five personality traits are—in the cases of Openness, Conscientiousness and Agreeableness—well-established and presumably have been underestimated while additional concordances were completely concealed due to the use of undifferentiated (or even grossly underspecified; see Alford and Hibbing, 2007) instruments. Of note, the inclusion of some specific measures may even inflate associations due to item overlap as, for example, Sibley et al. (2012) and Verhulst et al. (2012) discuss for the values subscale of the NEO-PI-R. But for most frequently used personality batteries this is clearly not a problem [see Gerber et al. (2011) for a discussion].

As a third caveat, although many of the cited studies strived to recruit international samples, there is a clear bias in favor of WEIRD (western, educated, industrialized, rich, developed; Henrich et al., 2010) countries, calling for high-powered thoroughly designed cross-cultural replication studies with a special focus on countries not sharing the traditional western alignment of conservative social and economic dimensions (e.g., post-communist countries). Of note, there is valuable work in this realm, generally supporting the view that associations between indicators of closed personality and cultural conservatism are quite stable whereas associations with the economic domain shift across cultural backgrounds in line with Johnston et al.'s reasoning (Malka et al., 2014; Roets et al., 2014; Fatke, 2017). However, presumably due to the extensive scope of these studies, methodic criticisms as outlined above apply here in a particular way (Ludeke and Larsen, 2017).

Importantly, fourth, even stable associations surely do not imply causality. Whether personality *causes* political orientation is a difficult question not answered definitely. Longitudinal studies finding the assumed causal pathway (e.g., Perry and Sibley, 2012) suffer from low power and representativeness. Others suggest that there is no causal relationship between both phenomena in either direction but their association traces back to a common genetic factor. However, these studies suffer from coarse measurements and usage of outdated instruments (Verhulst et al., 2012; Osborne and Sibley, 2020). Of note, a causal relationship is no prerequisite for evolutionary informed approaches as outlined below.

Lastly, yet, there is little research on potential moderators. While the relationships between personality and social political orientation are impressively stable, personality's association with economic preferences is sensitive to intercultural and interindividual differences as political engagement (see above), education, income and social class (Bakker, 2017; Federico and Malka, 2018; Furnham and Fenton-O'Creevy, 2018). While these factors are well-known in political sciences, rather psychological concepts as childhood trauma also seem to moderate the association between personality and political orientation (De Neve, 2015).

Summing up, the literature clearly indicates that associations between basic personality traits and political orientation are substantial. Dismissals of those are likely attributable to missing differentiations on the conceptual and methodical level.

## Political Orientation and Prosocial Behavior

Missing links between political orientation and prosocial behavioral inclinations in controlled experiments could hardly be reconciled with evolutionary models. However, initial evidence indeed yielded null-results for the most part: Fehr et al. (2003) reported greater behavioral indices of trust among proponents of Germany's largest parties but no association with political orientation *per se*. Likewise, Balliet et al. (2016) found no association of investments in the Trust-Game (TG; the trustee receives the tripled transfer of the investor and may or may not give back) and political orientation. Participants merely showed ingroup-favoritism by transferring more to fellow republicans vs. democrats. Alford and Hibbing (2007) found no relationship between political orientation and withdrawals from a common pool of funds and with offers in an Ultimatum-Game (UG; the first player needs to decide whether or not he gives a part of his endowment to the second player who has the option of rejecting the offer which leads to no payment to both players) and Anderson and Mellor (2005) did not find differences between participants with democratic vs. republican leanings and between liberal vs. conservative participants in averaged transfers in 30 rounds of the Public-Goods-Game (PGG; participants can invest in a public good, which is multiplied and evenly distributed across players at the end of a round) and the Trust-Game.

By contrast, Van Lange et al. (2012) and Chirumbolo et al. (2016) showed in multiple samples that people with competitive or individualist social value orientations (operationalized by a point-allocating game) are politically more conservative than people endorsing a prosocial orientation. Recently, Grünhage and Reuter (2020a) reported significant associations between conservatism and Cooperativeness (PGG) and Trust (TG), being specifically associated with SDO and RWA, respectively. Accordingly, both game scores were differentially associated with the approval of parties' policy stances: Trust predicted support for traditionalist vs. progressive parties, whereas Cooperativeness predicted accordance with market-liberal vs. regulation-focused parties. Furthermore, political orientation did not predict unspecific risk-tolerance operationalized by a lottery game.

We suggest that the inconsistency of results is due to two factors:

First, studies finding null-results allowed characteristics of the other player(s) to influence participant's decisions, potentially covering the small but meaningful effects of political orientation. Information about the opponent's political leanings (Balliet et al., 2016), their behavior in previous rounds (Anderson and Mellor, 2005), and potentially even mere resemblance with physically present opponents (cf. DeBruine, 2002) certainly are capable of overriding political orientation's influence on initial behavioral tendencies. If these situational factors are controlled, as done in the studies by Chirumbolo et al. (2016), Grünhage and Reuter (2020a), and Van Lange et al. (2012), an association of behavioral tendencies with political orientation is observable. However, such a view implies that behavioral dispositions may also be amplified by certain situational frames. In support of this assumption, Halali et al. (2018), for example, found SDO to be predictive of egoistic decisions in nested social dilemma games where the participant allocated money to himself, exclusively to the advantaged ingroup or universally to the in- and the disadvantaged outgroup. Across three intergroup-frames (Whites vs. Blacks, Christians vs. Muslims, and Israelis vs. Palestinians), SDO was consistently negatively related to universal allocations, positively associated with egoistic decisions, and unrelated to parochial cooperation.

Second, inconsistent patterns of relationships between political variables and behavior in very diverse games have been prematurely interpreted as pointing to missing relationships (Alford and Hibbing, 2007; Haesevoets et al., 2015). But, as Haesevoets et al. (2015) demonstrated intriguingly, behavior in various games is not intercorrelated very strongly, suggesting that different games assess different behavioral dispositions. A blatant example related to our first point is the difference between the Ultimatum- and the Dictator-Game (DG; the receiver has no option to reject the offer). Transfers in the former but not in the latter were found to be negatively associated with RWA and SDO (Haesevoets et al., 2015). However, in sharp contrast to the DG, the UG's structure allows perceived characteristics of the second mover to influence the first mover's decision massively and, thus, possibly, to override initial inclinations. In a similar vein, RWA only negligibly correlated with behavior in classic Prisoner's Dilemma arrangements but predicted behavior in the assurance variant (where the best outcome is yielded by mutual cooperation instead of defecting a cooperative partner). Hence, the critical question is, which game is most reflective of the behavioral disposition in question. Alford and Hibbing (2007) based their rejection of meaningful associations between interpersonal behavioral tendencies and political orientation on a DG and a simple pool withdrawal game with a clear modal choice, which—in addition to the mentioned caveats—introduces the statistical problem of variance restriction. Contrarily, withdrawals in a Commons Dilemma (where players can individually harvest from a collective asset that is doubled at the end and distributed equally across >2 players) were shown to be significantly associated with RWA and SDO (Haesevoets et al., 2015).

Hence, the emerging picture seems to be: The less characteristics of the opponent(s) can influence decisions, and the more ecologically valid game paradigms are, the stronger measures of political orientation or its psychological foundations predict concrete interpersonal behavior. Future research, thus, should apply well-validated paradigms to yield clearly defined measures of distinguishable aspects of pro- or antisocial behavior as Cooperativeness, Trust, Reciprocity, or greedy vs. anxious Defection (cf. De Dreu et al., 2010; Englmaier and Gebhardt, 2016). Furthermore, the moderating influences of the opponents' characteristics clearly deserve attention. These may not only reduce but also strengthen the predictive power of political orientation measures. Lastly, to our knowledge, there are no longitudinal studies explicitly examining the causal order of behavioral inclinations and political orientation.

## Integration and Discussion

Especially in light of recent evolutionary accounts of political orientation's roots, the question arises, why the existence of meaningful correlations of personality and interpersonal behavior with political orientation is questioned critically. We have outlined conceptual and methodological problems that may have covered meaningful, and indeed consistent, associations between personal, interpersonal, and political temperaments in the past and cited cumulating studies showing that these relationships exist.

Knowing these associations can further qualify evolutionary accounts on political orientation. Specifically, with regard to the framework offered by Claessens et al. (2020), it is important to consider the conspicuous “primacy” of the social or cultural domain as compared to the economic domain in political orientation's association with personality demonstrated by Johnston and colleagues (Johnston's et al., 2017; Johnston, 2018). Connecting lower scores on Openness and higher scores on the Politeness aspect of Agreeableness to increased group-binding motivations is intuitively plausible. Going further, we propose that in less group-oriented societies (i.e., among social liberals) variance in cooperativeness across larger networks is restricted, because here, individuals need to cooperate with and accordingly trust strangers to gain evolutionary benefits. Curry (2016) postulates mutualism/coordination and exchange as two domains of morality aimed at increasing benefits through cooperation (e.g., by showing ingroup favoritism in the first or establishing reciprocity norms in the second case). Possibly, conservatism and liberalism thus equal distinct adaptations to this evolutionary challenge. Note that this framework does not require personality and behavioral dispositions to causally precede political orientation. Flipping perspectives, such a framework may answer questions left open by Johnston's et al. (2017): as outlined above, these authors suggest that engaged people align their economic preferences to those of socially attractive political elites but they leave open why these elites stand for what they stand for economically. The findings summarized here suggest that the variance of Agreeableness and Cooperativeness is less restricted in conservative (group-oriented) than in liberal communities. In line with findings of higher assertiveness and lower withdrawal-tendencies in conservatives, it is thus more likely that assertive, uncompassionate, dominant and less universally cooperative individuals take the lead than in liberal communities where compassionate Agreeableness and Cooperativeness are distributed more equally. Of note, such a framework allows for unengaged conservative individuals to actually support liberal economic policies as Johnston's et al. (2017) showed. Furthermore, thinking along evolutionary lines may answer the question why the social domain dominates the political divide, which was not the case until the 1960s (Ellis and Stimson, 2012). We suggest that the significance of group-binding motifs is contingent on situational factors. Precisely, if, as for the largest part of our evolutionary past, human life is organized in small groups anyway, economic class conflict may prevail. But since globalization and digitalization massively expanded social networks, social conflict lines gained significance. Naturally, this model remains speculative and tentative at this point. Much more research on temporal, social, cultural, psychological, biological, and genetic influences should be integrated.

Hence, this review only scratches the surface. We intentionally focused on findings and perspectives not covered by extensive prior reviews (Jost, 2017; Jost et al., 2017), on one specific concept of personality and on one specific class of experimental paradigms, thereby dismissing a wealth of literature on dispositional needs, values, regulatory foci, biases etc.

Put simply, the reviewed literature suggests that people advocating correctly defined and thoroughly assessed right vs. left or conservative vs. liberal policies do differ in their psychological profiles with respect to personality and interpersonal behavioral dispositions. Presumably, this is because a proper definition of conservative vs. liberal refers to evolutionarily conserved differences in cooperativeness and group-mindedness (Claessens et al., 2020).

Knowing about the psychological peculiarities of right- vs. left-wing adherents is of particular relevance—unsurprisingly—in the political realm. Knowing the psychological and behavioral correlates of political orientation can enhance traceability of political decisions, and it may prove fruitful in the qualification of theories of political science: Especially, false assumptions of equivalence as implicated in extremism-, populism- or terrorism-accounts should be reevaluated in the light of the psychological peculiarities associated with left- vs. right-wing orientations (Baron and Jost, 2019).

Generally, an adequate, empirically supported appreciation of the mutual relationship between political convictions and psychological characteristics may help disenchant myths and—ultimately—improve inter-ideological dialogue.

## Author Contributions

TG contributed the first draft of the manuscript. MR revised the manuscript. All authors contributed to the article and approved the submitted version.

## Conflict of Interest

The authors declare that the research was conducted in the absence of any commercial or financial relationships that could be construed as a potential conflict of interest.
